# Family Physicians as Team Leaders: “Time” to Share the Care

**Published:** 2009-03-15

**Authors:** Kimberly S. H. Yarnall, Truls Østbye, Katrina M. Krause, Kathryn I. Pollak, Margaret Gradison, J. Lloyd Michener

**Affiliations:** Department of Community and Family Medicine, Duke University Medical Center; Duke University Medical Center, Durham, North Carolina; Duke University Medical Center, Durham, North Carolina; Duke University Medical Center, Durham, North Carolina; Duke University Medical Center, Durham, North Carolina; Duke University Medical Center, Durham, North Carolina

## Abstract

A major contributor to shortfalls in delivery of recommended health care services is lack of physician time. On the basis of recommendations from national clinical care guidelines for preventive services and chronic disease management, and including the time needed for acute concerns, sufficiently addressing the needs of a standard patient panel of 2,500 would require 21.7 hours per day. The problem of insufficient time indicates that primary care requires broad, fundamental changes. The creation of primary care teams that include members such as physician assistants, nurse practitioners, dietitians, health educators, and lay coaches is important to meeting patients' primary care needs.

## Introduction

Only approximately half of eligible patients receive recommended preventive, chronic disease, and acute care services (1). Inadequate office systems are often cited as limiting clinician efficiency and effectiveness (2). Physicians have reported barriers to delivering services that include external regulations, reimbursement structures, and lack of time (3).

Several interventions to improve preventive service delivery and chronic disease management have been tested. For example, Put Prevention Into Practice was an effort to reorganize the delivery of preventive services, but evaluations of this program failed to show significant sustained increases in preventive service delivery (4). Efforts to automate reminder systems and improve efficiency in both prevention and chronic disease management have yielded initial improvements in randomized trials (5), but the effectiveness of computerized prompts appears to drop rapidly in the 6 months after implementation (6).

The common denominator in the failure to deliver services is probably lack of physician time. Our previous analyses have suggested that primary care physicians simply do not have enough time to deliver all the preventive and chronic disease services recommended in national clinical care guidelines (7,8). To better understand the problem of insufficient time, we examine how family physicians allocate their time in the office among the types of service delivered (preventive, chronic care, or acute). We compare these data with published estimates of the time needed to adhere fully to recommendations for preventive service delivery and chronic disease management while still fulfilling existing acute care demands. We then discuss a delivery model for health service that has the potential to overcome the time demands on primary care clinicians.

## Analysis of Actual and Recommended Time for Patient Care

### Actual time distributions

To determine the distribution of family physicians' time spent providing care to patients, we analyzed data from the National Ambulatory Medical Care Survey (NAMCS) for 2003 (9). The NAMCS is a national survey of the provision of ambulatory medical care services, regardless of specialty, in the United States. The survey is based on a sample of visits to nonfederally employed, office-based physicians who are primarily engaged in direct patient care (9).

The NAMCS provides information about each visit sampled that includes the specialty of the provider (eg, family medicine, pediatrics), what type of provider was seen (eg, physician, nurse), the type of visit (acute, preventive, or chronic care), and the length of the visit in minutes. We selected only visits to family medicine physicians for our analysis.

We identified the proportion of visits with family physicians that were for acute care, preventive care, and chronic disease management (routine and acute flare-up). The NAMCS also uses the visit category "Pre-/Post-surgery," which makes up 1% of the visits; these were subsumed under acute care for this analysis.

The average time in minutes spent with the physician in each type of office visit was determined. On the basis of the proportions of the 3 types of visits and their respective lengths, we calculated the proportion of time spent on each type of care. These adjusted proportions were then applied to the number of hours per week that family physicians spend in direct patient care, 40.2 hours (10), to arrive at the average number of hours per day spent providing acute, chronic, and preventive care.

### Required time distribution

Our earlier reports (7,8) used studies of national preventive service and chronic disease management guidelines to estimate the total time required per year to deliver recommended care. The time was irrespective of the number or length of visits or the number of diseases dealt with in a visit. We used a theoretical patient panel of 2,500 (a typical panel size for family medicine [11]), with an age, sex, and disease prevalence representative of the US population. In the study on time for preventive services (7), we used published and estimated times per service to determine how long it would take to provide all services recommended by the US Preventive Services Task Force. In the study on chronic disease care (8), we applied 10 minutes per visit for the recommended number of yearly visits for 10 common chronic diseases, and we allotted additional visits for uncontrolled or severe cases if recommended by the guideline (8). The estimated times required to meet national guidelines also assumed a well-run, fully staffed practice with a functioning informatics system that provided patient information to the physician and staff. We did not consider in the calculations the amount of time spent on paperwork or contacting patients by telephone. To estimate the time required for acute care, we assumed it would remain the same as in current practice.

### Results

Table 1 presents the actual distribution of family physician time by type of visit: acute, chronic, or preventive. Almost half of visits are for acute problems, more than one-third are for chronic disease, and approximately 15% are for preventive services. Acute visits require an average of 17.3 minutes, less time than either preventive or chronic visits. After adjusting for the length of each type of visit, family physicians spend approximately 3.7 hours of their day in acute care (46%), 3.0 hours of their day in chronic disease care (38%), and 1.3 hours delivering preventive services (16%).

Table 2 presents the number of hours per day required to comply fully with current guideline recommendations for preventive services (7) and for the 10 most common chronic diseases (8). Time spent providing acute care is added to complete the estimate of total time needed to deliver all clinical care. Taken together, providing care for prevention, chronic care, and acute care to an average patient panel would require 21.7 hours a day.

## A Service Delivery Model to Meet Guidelines for Care

The time required to deliver recommended primary care is almost 3 times what is available per physician. To meet guidelines for chronic disease management and prevention, physicians would need to work 22-hour days and reorganize their practices so that they spent almost 50% of their time in chronic disease management and a third of their time in prevention. It is easy to see why there are shortfalls in service delivery; both the time required and the focus on urgent health issues rather than long-term health concerns prevent primary care clinicians from delivering recommended care.

The time calculations presented in this report assume ideal office work flow and information transfer and do not account for time on the phone or doing paperwork. Efforts to streamline care through better office organization and informatics may improve some other issues surrounding service delivery but will not address the core problem of inadequate time. It is unlikely that even the most organized office and most motivated staff can fulfill the care recommendations for all patients.

During the past decade, national organizations (2), professional associations (12,13), and health services researchers (14) have described US primary care as a system in crisis that must be fundamentally reorganized. Our findings reinforce these concerns. Although projections of the physician workforce consider numerous variables in complex equations, they rarely consider the simple additive effect of brief interventions administered to a large number of patients or the increasing prevalence of chronic disease. As medical knowledge advances, we can expect more screening tests, more behavioral risk factor counseling, and more services such as genetic testing to become available. The consequences of the obesity epidemic and the aging of the population will stress the current system further. As disease treatment and prevention options advance, the increasingly complex health data that must be processed and shared with patients in an understandable way will require longer or more frequent visits, or both.

### Implications for the health care system

Our data suggest that increasing the delivery of disease prevention and chronic disease management services will require a shift away from the traditional approach to service delivery — focused on acute care with chronic disease and preventive services as secondary considerations — to new models in which chronic disease management is a central consideration, with preventive efforts following closely in importance and time spent. Wagner and colleagues have argued for this transition to a chronic care model (15) and demonstrated that it can improve patient outcomes (16). Our data add another dimension to this argument. Acute care, although still an integral part of primary care, can no longer be the driving force behind the organization of the primary care clinician's office or schedule, or the major source of reimbursement.

In the future, physicians should spend most of their time in chronic disease management. This shift from acute to chronic care will require an important change in physician mindset, a retooling of office staff and information systems, and a change in reimbursement and insurer incentives to providers and patients. Electronic sharing of information will be needed to manage the increasing amounts of clinical data for each patient. Information systems should focus on longitudinal chronic disease and preventive care management, including systems to track the care of patients with diseases and to prevent errors.

### Alternatives for solo physicians

One current approach to care reduces the panel size for a primary care clinician to 1,000 (or fewer) patients. This approach allows the clinician time with each patient to focus on chronic disease and preventive care health issues while addressing acute concerns as needed. To adequately reimburse the physician, this model requires either many more but shorter visits during the year or patients to pay an additional retainer (usually $1,500 annually) for physician services. The system of more but shorter visits has been tried for preventive services delivered in 2 appointments with some success, but it has raised patient concerns regarding the increased number of copayments for services (17). Boutique practices requiring retainers represent a growing industry but recruit only a select group of physicians and patients who are willing to pay more out of pocket. From a national standpoint, either of these payment strategies reduces the pool of primary care clinicians available and could lead to widening disparities in health care.

### Team-based care

The most viable solution to the time problem facing primary care physicians, especially within the constraints of the current reimbursement system, is to extend the roles of nonphysician clinicians. Additional nonphysician clinicians — including physician assistants (PAs) and nurse practitioners (NPs) — can expand the amount of time available for patient care and allow physicians to focus on the most complex medical care issues. This collaboration can take many different forms depending on the patient population, patient desires, insurance reimbursement, and the needs and structure of the community.

For example, Duke University has created the Just for Us program to care for seniors who have complicated medical conditions and are living in clustered housing (18). This program provides chronic disease management, some preventive care (eg, influenza vaccination), and limited acute care in the home. A health care team is led by a physician specializing in geriatrics and includes PAs (who bill for their services through the local federally qualified health center [FQHC]), social workers (who are employees of county social services), case workers (paid for by the Just for Us program and by the insurer, North Carolina's Medicaid program), a dietitian (paid through private insurance and Medicaid), and an occupational therapist (funded by a foundation grant) who evaluates fall risks in the home. Medical care is coordinated with the patient's primary care physician to reduce the number of issues that need to be dealt with in traditional office visits.

### Teams in the traditional office

In a traditional office-based practice, a team of providers would care for patients under the leadership of a primary care physician. This model would implement the Future of Family Medicine recommendations for a medical home, including patient-centered care, continuity of care, and same-day access (12). It would differ by having PAs and NPs provide the bulk of the services, becoming the primary providers for most patients in the practice. In this team practice, PAs and NPs would provide acute care (through open access or urgent care within the practice), preventive care, and some chronic disease management. Primary care physicians would see patients who were the sickest or had the most complicated illnesses and spend the rest of their time leading the team of health care providers to oversee, provide advice, and coordinate comprehensive care for the patient panel. Two full-time PAs or NPs and 1 supervising physician seeing patients could meet the time requirement to care for a panel of 2,500 patients. Additional PAs or NPs per physician would allow for care for a larger panel of patients while freeing the physician for team leadership and administrative work monitoring the practice's progress toward disease management goals for its patients.

Several studies have demonstrated patient satisfaction with midlevel providers (19,20). In addition, a recent study of Midwestern family practices showed that team-based practices using midlevel providers improved preventive screening rates. In that study, rural practices using PAs to see patients for acute and preventive care visits had the highest rates of preventive screening, nearly 90% (21). Similarly, Duke University has opened 2 small practices in collaboration with the FQHC to increase the availability of primary care for underinsured and uninsured patients. Ninety percent of visits are conducted by 3 PAs under the supervision of a part-time physician (22).

Adding other nonphysician clinicians to the practice — including dietitians, pharmacists, social workers, case workers, and occupational or physical therapists — can expand the scope of care available for patients by focusing on exercise and healthy eating habits in the context of their disease (availability would be based on billing and insurer reimbursement) (23,24). Including these providers would allow practices to move beyond the clinical encounter as the central focus for care delivery. Potential care could then include reimbursed group visits, patient-directed self-management teaching, case management, and educational home visits (23). Pilot programs for such strategies have been developed in some parts of the country, some by insurers and others by academic medical centers and large practices (25,26). Reimbursement strategies are possible even within the current payment system (27). Communication using e-mail and the Internet may also enhance medical care but changes in reimbursement for such activities will be required for these types of communications to expand and thrive.

### Involving the community

Extending beyond the practice walls and involving the community has a greater potential to change health behaviors than the traditional doctor visit. Most changes in health behaviors occur as communities reset what constitutes acceptable behavior — for example, the change in public sentiment regarding smoking and secondhand smoke, and the subsequent designation of smokefree environments. Engaging the community to improve health requires collaboration with health departments, employers, and community leaders such as ministers and politicians. This engagement can also lead to the development of resources such as community exercise areas and nutrition classes in workplaces and churches. Primary care physicians can best serve these community endeavors by functioning as collaborators and advisors. Community collaboration has the potential to change health behavior while reorienting attitudes toward the importance of disease prevention and management, and to begin bridging the historic divide between medical care and public health.

## Conclusion

There are not enough primary care physicians to meet the recommended care guidelines within the current model of a single physician providing all required preventive, chronic disease, and acute care to patients in his or her practice. As the number of guidelines and tests increase, as the population ages, and as chronic disease rates increase, the current model of health care delivery will be further strained. New models of team and community care can provide solutions to the problem of time, even within the current reimbursement system. Studies of new practice models and community engagement are needed to evaluate how well they provide the recommended care and to answer the larger question of whether fully implementing the available recommendations for preventive and chronic disease care can truly improve the health of the population.

## Figures and Tables

**Table 1:** Time Spent by Family Physicians to Care for Patients, by Type of Visit, United States, 2003

**Type of Visit**	% of Total Visits^a^	Mean Length of Visits (Minutes)^a^	% of Clinical Time	Hours/Week	Hours/Day
**Acute**	49.3	17.3	45.8	18.4	3.7
**Chronic**	36.1	19.3	37.4	15.0	3.0
**Preventive**	14.6	21.4	16.8	6.8	1.3
**Total or mean **	100.0	18.6	100.0	40.2^b^	8.0

a Data obtained from National Ambulatory Medical Care Survey (9).

b Source: American Academy of Family Physicians (10).

**Table 2:** Time Required to Meet Current Clinical Guideline Recommendations

**Type of Visit**	Hours/Day	Hours/Week	% of Clinical Time
**Acute**	3.7^a^	18.4	17.0
**Chronic**	10.6^b^	53.0	48.9
**Preventive**	7.4^c^	37.0	34.1
**Total**	21.7	108.4	100.0

a Calculated in Table 1.

b Source: Østbye et al (8).

c Source: Yarnall et al (7).
